# Assessing cell survival and biosignature preservation of the extremely thermoacidophilic archaeon *Acidianus manzaensis* after exposure to Mars-like conditions

**DOI:** 10.1093/femsmc/xtag043

**Published:** 2026-07-30

**Authors:** Sebastian V Gfellner, Janina Groninga, Cyril Colas, Guillaume Gabant, Andreas Lorek, Stephen Garland, Mickael Baqué

**Affiliations:** UPR4301 Center for Molecular Biophysics (CBM), University of Orléans, 45071 Orléans Cedex 2, France; MARUM—Center for Marine Environmental Sciences, University of Bremen, 28334 Bremen, Germany; UPR4301 Center for Molecular Biophysics (CBM), University of Orléans, 45071 Orléans Cedex 2, France; UMR7311 Institute of Organic and Analytical Chemistry (ICOA), University of Orléans, 45067 Orléans Cedex 2, France; UPR4301 Center for Molecular Biophysics (CBM), University of Orléans, 45071 Orléans Cedex 2, France; Institute of Space Research, German Aerospace Center (DLR), 12489 Berlin, Germany; Institute of Space Research, German Aerospace Center (DLR), 12489 Berlin, Germany; Institute of Space Research, German Aerospace Center (DLR), 12489 Berlin, Germany

**Keywords:** Mars simulation chamber, exposure experiment, UV-irradiation, metabolomics, biomarkers, astrobiology

## Abstract

Considering the harsh surface conditions on Mars, terrestrial organisms that can survive and remain detectable after exposure to similar conditions provide invaluable models for guiding current and future search-for-life missions. To this end, we evaluated the extremophilic archaeon *Acidianus manzaensis* grown on ESA01-E Mars analog material as a model organism. We exposed cell–mineral mixtures to one month of desiccation and 2 weeks of Mars-like conditions in a Mars simulation chamber to evaluate the potential for cell survival after exposure to these conditions. In the search for reliable biomarkers in other planetary environments, such as Mars, molecules that are stable over geological timescales while preserving information indicative of their potential biological origin are crucial. Thiophene-bearing quinones fulfill these requirements, and thiophenes, which are their basic moieties, have been discovered on Mars. Therefore, we analysed the thiophene-bearing quinone composition of *A. manzaensis* using mass spectrometry-based metabolomics and discussed potential molecular alterations. Successful recultivation after 1 month of desiccation and 2 weeks of exposure to Mars-like conditions proved the durability of the organism and its capability for cell recovery after exposure to extreme conditions, paving the way for further investigation.

## Teaser

We evaluated the capacity of microorganisms to withstand Mars-like conditions by exposing them to a Mars simulation chamber and investigated potential biosignatures for astrobiological life detection.

## Introduction

More than 60 years of planetary exploration have shown evidence that the early planetary stages of Earth and Mars shared similar environmental conditions, including the presence of liquid water and volcanic activity on the surface, coupled with an atmosphere rich in carbon dioxide (CO_2_) (e.g. Pollack et al. 1987, Wordsworth 2016). Whether these conditions lasted long enough to lead to the origin of life on Mars is an overarching question in astrobiology and the main focus of current and future robotic missions, such as Mars2020, ExoMars, and Mars Sample Return missions (Vago et al. 2017, Williford et al. 2018, Beaty et al. 2019). However, in an Archean early Earth analogous to a Noachian early Mars setting (∼4 Ga ago), one must consider the surface and subsurface environments at that time and the interplay between geo- and potential biospheres in a pervasive volcanic and hydrothermal setting (Carr and Head 2010, Arndt and Nisbet 2012, Grosch and Hazen 2015). Chemolithoautotrophic organisms, as found in the most ancient terrestrial paleorecords, are among the best analogs for putative early Martian life (Westall et al. 2013, Lyons et al. 2024). Indeed, because they use CO_2_ as a carbon source and redox-alter minerals by oxidizing inorganic compounds, such as iron, sulfur, and other reduced inorganic sulfur compounds, all of which are found on Mars (Lewis et al. 2018, Millan et al. 2022), their metabolic pathways make them excellent candidates for astrobiological research. Heat- and acid-loving thermoacidophilic microorganisms are analogs of the potential life forms of such planetary stages (Lewis et al. 2018). In particular, these microorganisms serve as excellent analogs for early Mars because of their physiology and capacity to tolerate the intense volcanic heat and acidic gases that were prevalent during the geochemically active Martian past (Lewis et al. 2018, 2021). Among these, the extremely thermoacidophilic archaeon *Acidianus manzaensis* from the order Sulfolobales was isolated from a hot fumarole in Manza, Japan (Yoshida et al. 2006). It thrives at high temperatures and low pH and tolerates high concentrations of heavy metals. Moreover, its genome has been fully sequenced, facilitating molecular studies of its metabolic capacity.

Isoprenoid quinones are of particular interest for investigating the potential molecular biosignatures of these organisms. Isoprenoid quinones are integral components of the membranes of all living organisms (Hiraishi 1999). These molecules consist of a hydrophilic head group and a nonpolar isoprenoid side chain, which confer amphiphilic characteristics that enable their integration into lipid bilayers. Their primary role is to act as electron and proton carriers in the electron transport chains of photosynthesis and respiration; they also serve as antioxidants (Hiraishi 1999, Nowicka and Kruk 2010). Quinone oxidoreductases transfer electrons to terminal oxidase complexes, which help maintain intracellular pH and generate a proton motive force through the reduction of caldariella- or sulfolobus-type quinones (Auernik and Kelly 2008). Thiophenes, benzothiophenes, and benzoquinones are the basic units of the headgroups of isoprenoid quinones (Owton 1999). Thiophene molecular fragments occur in 2.72 Ga-old stromatolites (Lepot et al. 2009), sedimentary structures formed by microorganisms that are among the oldest fossils on Earth (van Kranendonk et al. 2008), and have also been found in association with anoxygenic phototrophic biofilms in 3.3 Ga sediments (Westall et al. 2011). Furthermore, sulfur compounds, such as ethanethiol and thiophenes, have been detected at the Gale Crater on Mars by the Curiosity rover, preserved in sediments older than 3 Ga, whose possible origins may be from diagenesis or pyrolysis of biological material, as well as abiotic origins (Eigenbrode et al. 2018, Millan et al. 2022). Abiotic thiophenes produced by thermal and/or aqueous alterations have also been observed in meteorites (Sephton 2002, Sephton et al. 2006, Ehrenfreund and Septhon 2006). They have also been formed abiotically under simulated volcanic and hydrothermal conditions, such as those that occurred on the early Earth and potentially on early Mars (Geisberger et al. 2021). These compounds exhibit significant stability and longevity and persist over geological timescales (Westall et al. 2011, Eigenbrode et al. 2018, Heinz and Schulze-Makuch 2020, Geisberger et al. 2021). Therefore, thiophene-bearing quinones may be valuable for astrobiological life detection.

The quinone composition of the order Sulfolobales consists of oxidized caldariellaquinones (CQ), sulfolobusquinones (SQ), and benzodithiophenequinones (BDTQ). Members of the Sulfolobales order exhibit varying compositions of saturated quinones in response to redox environments. Consequently, the quinone profile of a specific Sulfolobales member can be used to reconstruct environmental redox conditions (Brassell et al. 1986, Hiraishi 1999, Elling et al. 2016, Becker et al. 2018, Elling et al. 2025). Nevertheless, the proportion of quinones varies among different Sulfolobales species, and adaptations to environmental conditions may be reflected in the distribution of SQs, CQs, and BDTQs (Elling et al. 2016). Caldariellaquinones are produced by organisms that thrive in harsh conditions (pH 1.4–2.6; 75°C–89°C) (Rosa et al. 1977), with a robust membrane structure observed in Sulfolobus and Acidianus species (de Rosa and Gambacorta 1988). Later, caldariellaquinones were found in *Sulfolobus solfataricus* and subsequently in *Metallosphaera sedula* (de Rosa et al. 1983a, b, Lanzotti et al. 1986, Huber et al. 1989), whereas benzodithiophenequinones were detected in *S. solfataricus* (Collins and Langworthy 1983, Trincone et al. 1986, 1992, Lanzotti et al. 1986). Variations in the CQ, SQ, and BDTG molecules produced are linked to the presence of oxygen during growth (Trincone et al. 1989, Nicolaus et al. 1992). For the Sulfolobales order, Elling et al. (2016) reported the distribution of CQ_6:0_ (86.1%) and CQ_6:1_ (12.2%) in *Sulfolobus acidocaldarius*, CQ_6:0_ (85.8%) and CQ_6:1_ (13.7%) in *S. solfataricus*, and SQ_6:0_ (42.9%), CQ_6:0_ (36.4%), and CQ_6:1_ (14.6%) in *Sulfolobus islandicus* as the main quinone components, with BDTQ_6:0_ (0.4%) only found in *S. islandicus*. Additionally, Gfellner et al. (2025) reported a distribution of SQ_4:0_ (2.7), SQ_4:1_ (66.7), SQ_5:0_ (8.8), SQ_5:1_ (5.5), CQ_4:1_ (2.6), CQ_5:1_ (6.9), and BDTQ_5:0_ (6.8) in *M. sedula*. To date, the distribution of quinones in *A. manzaensis* has not yet been determined.

However, quinone distribution may vary under extreme environmental conditions. In particular, the harsh conditions of the Martian surface must be considered when examining not only the potential survival of microorganisms but also the preservation of these compounds. Studies have been conducted with radiation-resistant acidophilic chemolithoautotrophic bacteria to determine their survival capabilities under simulated Martian environmental conditions, such as atmosphere, pressure, temperature, and UV radiation (Gómez et al. 2010). Additionally, the effects of mineral shielding on the preservation of biosignatures have been widely discussed (e.g. Westall et al. 2015, Bosak et al. 2021), particularly when examining the damaging effects of UV radiation on organic molecules shielded by rocks and minerals (Carrier et al. 2019). In this study, we focused on the effects of extreme desiccation and radiation on the survival of *A. manzaensis* and the formation of potential biosignatures in the presence of mineral materials and under Martian conditions (temperature and pressure).

Hence, *A. manzaensis* was cultivated on the Mars analog material ESA01-E, which is known for its physicochemical similarity to Martian basalt. A dehydration experiment was conducted over a period of 1 month to test the survivability of the species under desiccated conditions. In this process, concentrated cell pellets of *A. manzaensis* were deposited into metal exposure wells to monitor cell survival upon recovery of the dried pellets in a strain-specific autotrophic medium (Kölbl et al. 2022) supplemented with tryptone as a nitrogen source (Yoshida et al. 2006). Furthermore, metabolomic analysis was conducted using ultra-high-performance liquid chromatography coupled with high-resolution mass spectrometry (UHPLC—HRMS) to assess the thiophene-bearing quinone distribution of *A. manzaensis* and present theoretical considerations of using them as potential biomarkers for life detection on Mars.

## Materials and methods

### Experimental design

**Figure 1:**
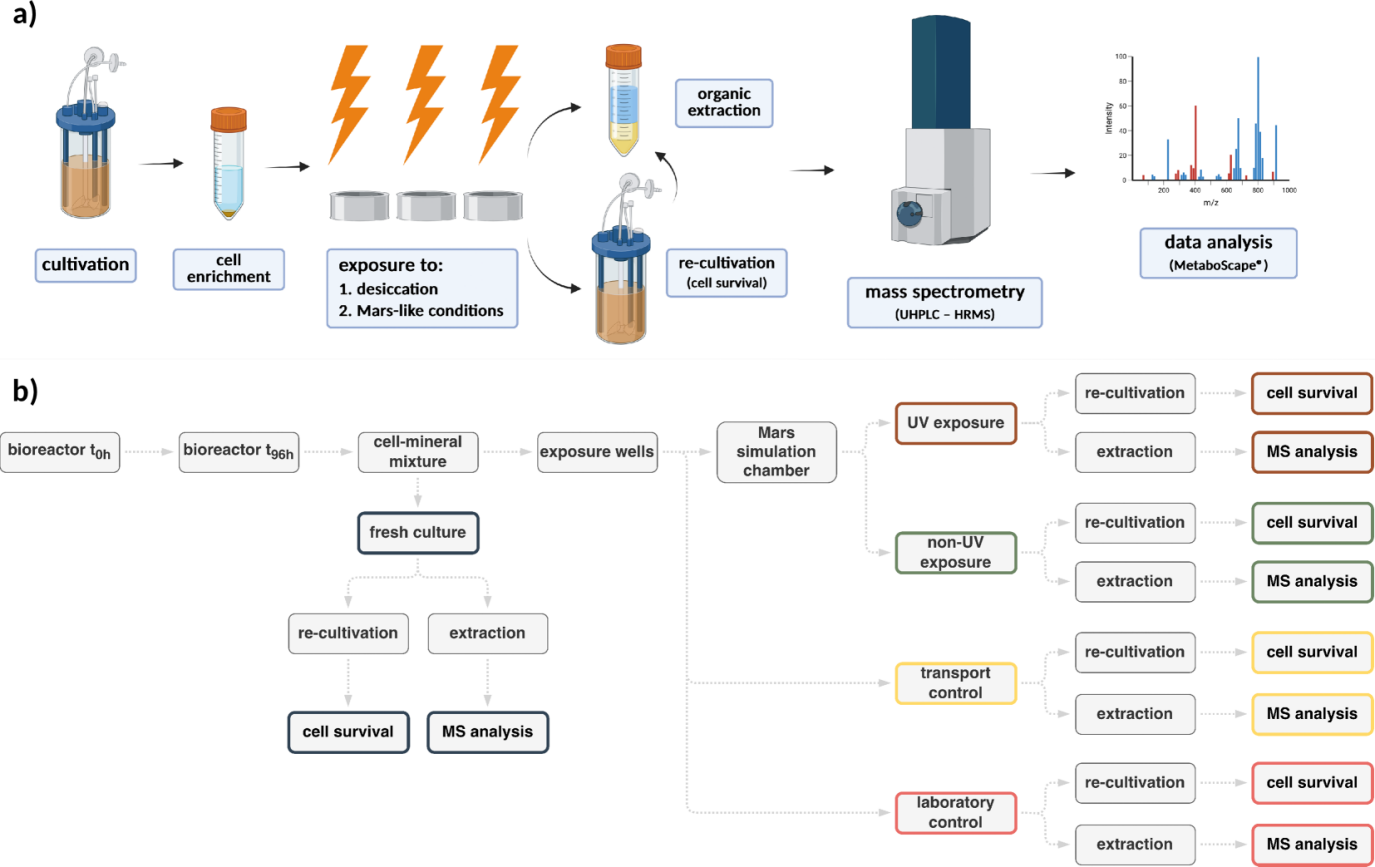
Overview of the workflow and experimental design. (a) Workflow exposure experiment: cultivation over a duration of 96 h, cell enrichment, and deposition of cell–mineral mixtures in exposure wells to conduct (1) desiccation experiment and (2) exposure to Mars-like conditions. The recultivation of the exposed cells was performed over a duration of 72 h to determine cell survival, followed by organic extraction, mass spectrometry analysis (UHPLC—HRMS), and data analysis (MetaboScape®) to investigate potential changes in the composition of thiophene-bearing quinones. Created with BioRender.com. (b) Experimental design of the exposure experiment from cultivation to sample analysis and determination of cell survival by recultivation of exposed and nonexposed cell–mineral mixtures. Extractions were conducted immediately after harvesting the cell–mineral mixtures of the fresh culture, followed by extraction and MS analysis of the samples in the Mars simulation chamber exposed to UV, nonexposed to UV, transport control, and laboratory control.

The experimental setup (Fig. 1A) included exposure of *A. manzaensis* cells deposited in metal exposure wells using the improved extraction and analysis protocol by Gfellner et al. (2025) to identify potential changes in the composition of thiophene-bearing quinones. The desiccation experiment was conducted with a separate cell–mineral mixture of a bioreactor following the same cultivation and extraction parameters as the exposure experiment, 1 month prior to exposure to Mars-like conditions. The experimental design of the exposure experiment is shown in Fig. 1(B). In brief, after cultivation for 96 h, cell–mineral mixtures were divided into four sets in order to compare the effects on cell recovery and mass spectrometry measurements of each step: fresh culture (Fc) served as the basis. Laboratory controls (Lc) were kept under controlled conditions (shielded from light in aluminum and air-sealed in a storage container under a laminar flow box at the CBM-CNRS Orléans), whereas the transport controls (Tc) were transported with sets dedicated to the Mars simulation facility. Owing to laboratory constraints, samples exposed to UV (e) and laboratory controls were recultivated together for growth comparison, and while kept under controlled conditions, samples exposed without UV (ne) and transport controls were recultivated at the same time 1 week later. Extraction was conducted continuously, while the remaining samples were stored at 4°C. Cell survival was determined by successful recultivation of microbial cells after desiccation and exposure to Mars-like conditions.

Mars analog material: The ESA Mars basalt analog material (ESA-01-E) from the European Space Agency Exploration Sample Analog Collection (ESA^2^C) (Smith et al. 2018) was retrieved from the International Space Analog Rockstore (ISAR) in Orléans, France. The original sample material was collected from Craig’s Quarry in County Antrim, Northern Ireland. The mineralogical composition was evaluated as follows: plagioclase (65.9 wt. %), olivine (14.5 wt.%), pyroxene (15.5 wt.%), and clay (4.1 wt.%). The elemental composition of ESA-01-E (Table 1) closely corresponds chemically and mineralogically to known Martian rocks selected by the ESA collection (Bost et al. 2013), and was used and well characterized in previous experiments (Foucher et al. 2022).

**Table 1:** Elemental composition of the ESA Mars basalt analog material (ESA-01-E). The data were obtained from Foucher et al. (2022).

Sample		ICP-OES data (mass %)										
ISAR reference	Type of rock	SiO_2_	Al_2_O_3_	Fe_2_O_3_	MnO	MgO	CaO	Na_2_O	K_2_O	Na_2_O + K_2_O	TiO_2_	P_2_O_5_
ESA-01-E	Picrobasalt	44.18	16.03	14.46	0.19	7.71	8.72	2.75	0.18	2.93	1.66	0.16

Microbial cultivation and sample preparation: *A. manzaensis* (NBRC 100595) was cultivated aerobically in a 1 l glassblower modified Schott-bottle bioreactor (Duran DWK Life Sciences GmbH, Wertheim/Main, Germany) with culture medium and Mars analog material (ESA01-E basalt, 10 g/l). ESA01-E basalt was manually ground using a hand grinder to obtain particles with diameters of 63–100 μm for optimal microbial growth, controlled by 63 μm and 100 μm mesh sieves with a 75:25% distribution of 63–100 μm, and baked overnight at 180°C. The cultures were previously adapted to the mineral material in three cycles, following the same cultivation procedure, and the stock cultures were stored at −80°C in a mixture of 50% glycerol and culture medium (50, 50, v:v). Cultivation was conducted using *A. manzaensis* specific medium consisting of 0.132 g/l (NH_4_)_2_SO_4_, 0.041 g/l KH_2_PO_4_, 0.49 g/l MgSO_4_ × 7H_2_O, 0.009 g/l CaCl_2_ × 2H_2_O, and 0.052 g/l KCl, and Allen trace element solution (0.91 mM MnCl_2_ × 4H_2_O, 1.18 mM Na_2_B_4_O_7_ × 10H_2_O, 0.08 mM ZnSO_4_ × 7H_2_O, 0.03 mM CuCl_2_ × 2H_2_O, 0.01 mM Na_2_MoO_4_ × 2H_2_O, 0.02 mM VOSO_4_ × 2H_2_O, and 3.56 μM CoSO_4_ × 7H_2_O) following Kölbl et al. (2022) with tryptone (0.1%) as N-source over a period of 96 h. The pH was adjusted to 2.0 using 5 M H_2_SO_4_. The bioreactor was maintained at a constant temperature of 73°C with steady stirring, and a continuous flow of CO_2_ at a total rate of 0.3 l/min (normalized to 1 atm and 0°C) was ensured. To monitor microbial growth, the culture was sampled continuously during the growth phase, and the cells were counted visually under a microscope (Olympus BX51 equipped with a Pixelink M20C-CYL camera) using a Neubauer Chamber (Carl Roth GmbH & Co. KG, Karlsruhe, Germany) and harvested upon reaching the stationary phase. Harvesting was performed by centrifugation in sterile 50 ml Falcon tubes at 3220 × *g* for 40 min. After harvesting, 800 ml of cell–mineral material was concentrated by a factor of 20 through centrifugation (3220 × *g* for 40 min). The supernatant was continuously removed, and the cell–mineral material was homogenized in a sterile 50 ml Falcon tube with additional fresh medium to a total volume of 40 ml, and stored at 4°C for 72 h before deposition in the metal exposure wells. The deposition was performed by pipetting 300 µl into each metal exposure well and continuously evaporating to a total volume of 2 ml. The samples were stored at room temperature before and during transportation to and from the exposure facility.

Desiccation experiment: Prior to exposure to Mars-like conditions, test samples were subjected to prolonged desiccation for 1 month. Concentrated cell–mineral materials deposited in the metal exposure wells were air-dried completely, desiccated, and stored in a fume hood at room temperature under oxic laboratory conditions and under atmospheric pressure for 1 month. The samples were wrapped in sterilized aluminum foil, which is critical because many lipids and quinones are highly photosensitive, and shielding them from ambient light is essential to avoid biomarker degradation during storage. Thereafter, six samples (A_1–3_ and B_1–3_) were transferred into 50 ml glass flasks for recultivation under the same conditions and tested for statistical significance as described for the exposure experiment below.

**Figure 2:**
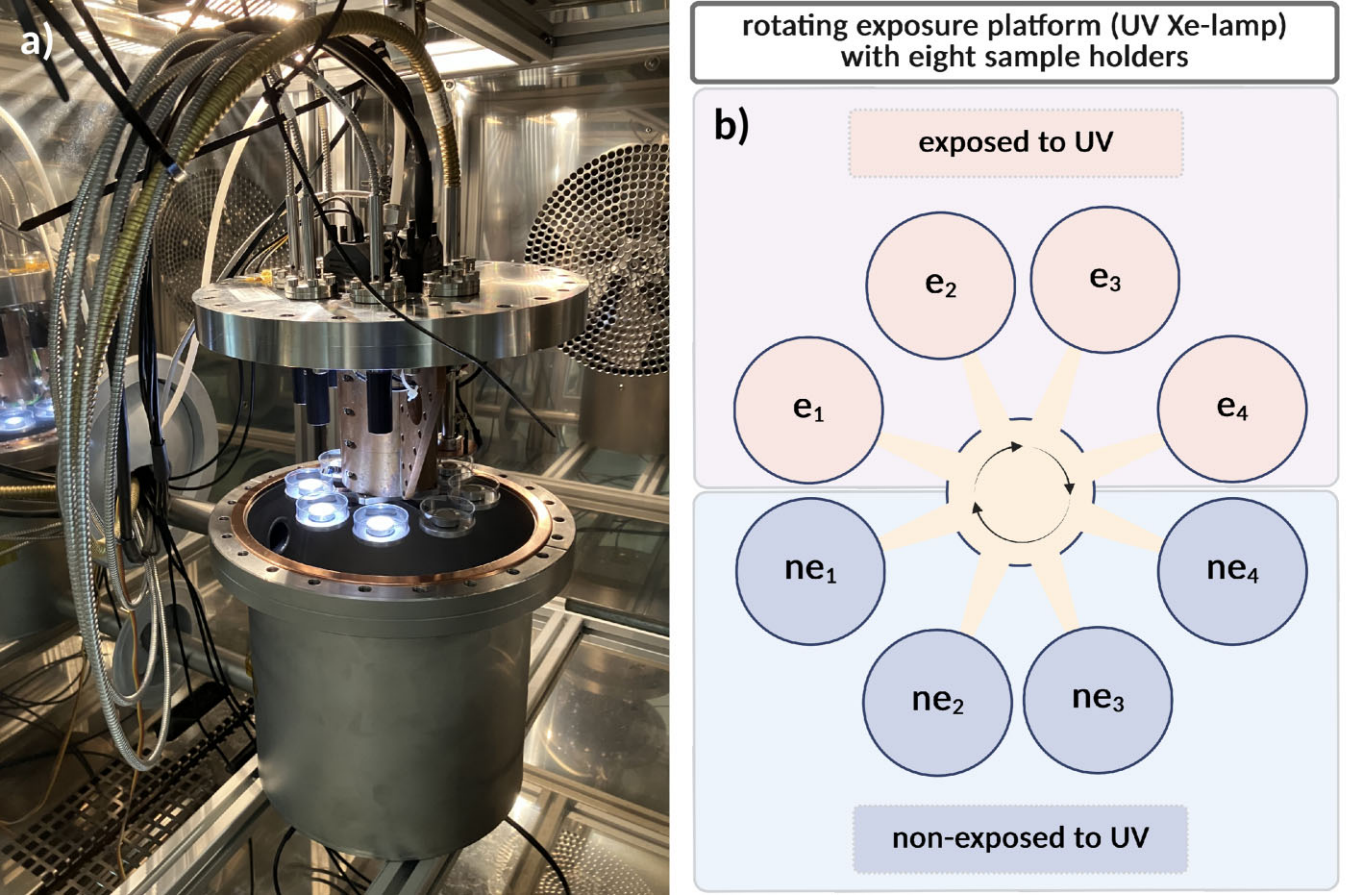
Mars Simulation Facility. (a) PASLAB at DLR Berlin with an open MSF chamber. (b) Detailed sketch of the exposure platform: exposure (e_1–4_) to UV light; no exposure (ne_1–4_) to UV light. Created with BioRender.com.

Exposure experiment: The exposure experiment was conducted with samples e and ne in the Mars Simulation Facility (MSF) at the Planetary Analogue Simulation Laboratory (PASLAB, DLR Berlin), Tc and Lc as controls, and Fc as a baseline. The MSF consists of a climate chamber (ATT Umweltsimulation GmbH), which contains a 10.3 l vacuum-sealed stainless-steel vessel (Fig. 2A) and a rotating platform with eight round aluminum sample holders. However, for the experiment conducted, the platform was not rotated, but the UV light at the illuminated side was turned on and off to simulate the day/night cycle. The experimental chamber has electrical connectors, inlet/outlet gas connectors, and four optical fibers for UV light provided by a 150 W Xenon lamp (Fig. 2B). Additionally, the experiment chamber is equipped with two SHT75 sensors (Sensirion AG, Switzerland) integrated with two Pt-100 temperature sensors to measure temperature and humidity (day-to-night: temperature of +15 to −50°C and relative humidity of 0% to 75%), calibrated for the Martian atmosphere. The gas flow through the experiment chamber is generated by a gas mixing system, and the inside pressure is controlled by a membrane vacuum pump (MV10Vario, Vacuubrand GmbH). The control of the whole system is based on a DAQ-system device (National Instruments Corp., USA) and Labview software (Lorek and Koncz 2013, Lorenz et al. 2023). The pressure was gradually decreased to 8 hPa, with a simulated Martian atmosphere composition of 95% CO_2_ and 5% air (4% N_2_ and 1% O_2_). The irradiance simulated the complete Martian solar spectrum (200–2200 nm) of the mid-latitude summer Sun’s diurnal cycle [nighttime (8 h) and daytime (16 h)] (de Vera et al. 2014, de la Torre Noetzel et al. 2018). Fourteen days of irradiation resulted in a total dose per sample of 12 029 kJ/m² and a flux of 14 kW/m².

Recultivation of exposed and non-exposed cultures: Upon arrival of the samples from the exposure facility, those deposited in the metal exposure wells were split in half using sterile scalpels under sterile conditions in a laminar flow hood. One-half of the sample was processed for organic extraction, and the other half was left in the metal sample holder and stored at room temperature under sterile conditions in a laminar flow hood until recultivation. *Acidianus manzaensis* was recultivated according to the protocol described by Kölbl et al. (2020). Recultivation was performed for each condition in four replicates: initial fresh culture (Fc), exposed to UV (e), nonexposed to UV (ne), transport control (Tc), and laboratory control (Lc). Cultivation was conducted in a shake incubator set at 75°C and 100 rpm over a period of 72 h in 50 ml glass flasks containing an equivalent of 1 ml concentrated cell–mineral material and 20 ml of *A. manzaensis* specific medium, including ESA01-E basalt and tryptone, as previously described. The 72 h period was chosen based on continuous cell motility to facilitate cell counting and doubling of cell density. To monitor cell growth, 1 ml aliquots of all samples were taken at 24 h intervals under sterile conditions in a laminar flow hood and counted in triplicate (for the desiccation experiment at t_0h_ and t_72h_; t_24h_ and t_48h_ were counted in single) and quadruplicate (for the exposure experiment t_0h_ to t_72h_) under a microscope using a hemocytometer (Neubauer chamber). The distinction between cells and mineral particles was conducted visually, considering cell movement, cell size (∼ 1 µm), and refractive index of mineral particles. Recultivation was visualized in a box plot (box-and-whisker plot) using BoxPlotR, representing not only the summary statistics of the growth curves (cells/ml over time) but also the distribution of the individual data points. It displays the ranges, minimum, lower quartile (Q1), median (Q2), upper quartile (Q3), and maximum values (Spitzer et al. 2014). Single-factor ANOVA analysis for determining the statistical significance of the endpoints of recultivation was performed using the LibreOffice extension Analysis of Variance (ANOVA) for all recultivated samples for the parameter cell/ml at *t* = 72 h for triplicate sets (desiccation experiment: A_1-3_, and B_1-3_) or quadruplet sets (Fc and exposure experiment: e, ne, Tc, and Lc) with α = 0.05.

### UHPLC—HRMS analysis

Semiquantitative analyses were conducted in reverse-phase liquid chromatography (RPLC) with mass spectrometric detection using ESI in positive mode. Chromatographic separations were performed on an Ultimate 3000RSLC system (Dionex, Germering, Germany) using an Acquity UPLC BEH C18 1.7 µm 2.1 × 100 mm column (Waters, Saint-Quentin-en-Yvelines, France). The column was heated at 60°C and the following solvents were used at a flow rate of 500 µl/min: H_2_O with 0.1% formic acid as solvent A and a mixture of methanol and isopropanol (50:50, v/v) with 0.1% formic acid as solvent B. Linear gradient elution was as follows: 0–2.5 min 3% B, 6% B at 4 min, 85% B at 13 min, a plateau at 100% B from 13.5 to 19 min and reequilibration from 19.1 to 23 min at 3% B. 2 µl of the organic extract were injected. Mass spectra were acquired on a maXis Q-TOF (Bruker Daltonik, Bremen, Germany) at a frequency of 1 Hz in the 50–1650 m/z range for MS experiments. The ESI source parameters were as follows: nebulizing gas, 2 bar; drying gas, 200°C at a flow rate of 9 l/min; capillary voltage, 4500 V. For MS/MS experiment in Data Dependent Acquisition (DDA) mode, the same parameters were used except the acquisition frequency: 2 Hz. Two precursors ions were selected per cycle into the 445 to 950 m/z range with exclusion after one spectrum for 0.07 min. Lock mass calibration was performed using ESI-TOF tuning mix (Agilent, Palo Alto, CA, USA) and lock masses at m/z 299.2945 [hexakis(1H,1H,4H-hexafluorobutyloxy)phosphazine; CAS #: 186406–47-3] and 1221.9906 [methyl stearate, CAS #: 112–61-8] for calibration correction and was applied to all MS and MS/MS spectra.

Feature detection: Molecular feature detection in UHPLC-HRMS measurements was performed using the Time-aligned Region Complete Extraction (T-ReX) 3D® feature-finder algorithm of MetaboScape 2024® (Bruker Daltonik GmbH, Bremen, Germany). For peak picking, a minimum intensity threshold of 5000 cts and a minimum peak length of four spectra across an m/z range of 250–2000 Da were set. The recursive feature-finding tool was deactivated and prior filtering ensured that only the features present in at least three measurements were retained. The primary ion was set to [M + H]+, with [M + NH_4_]+ and [M + Na]+ as the seed ions. When applicable, the adducts and doubly charged ions were grouped based on an ion deconvolution threshold of 0.8. Quinone annotation was performed utilizing a comprehensive target list of potentially occurring sulfur-bearing quinones, adhering to a maximum m/z tolerance of 2.5 ppm, an mSigma value of 40 (narrow) to 250 (wide), and an MS/MS similarity score of > 800, if MS/MS spectra were present. The extracted ion chromatograms (EIC) of the annotated compounds were reviewed, and anomalous peaks were excluded.

## Results and discussion

### Desiccation experiment

**Figure 3:**
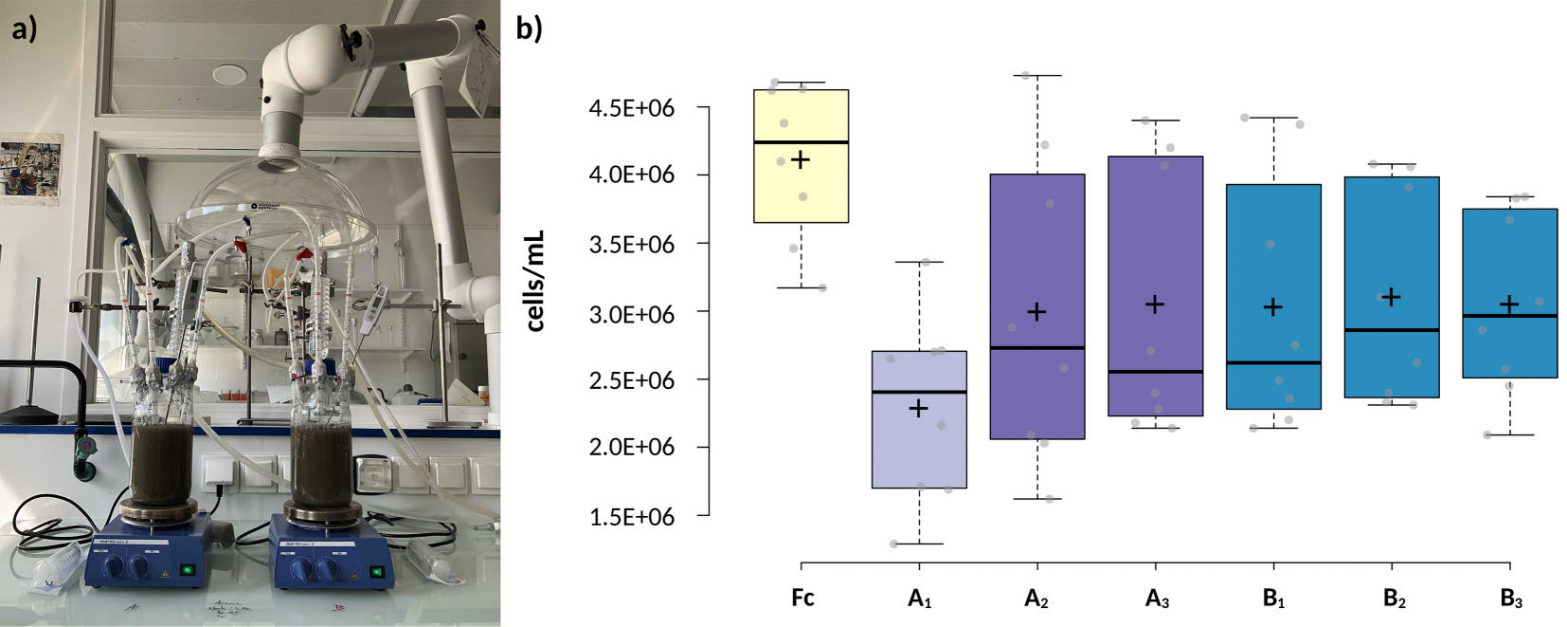
Bioreactors and recultivation of desiccated cells. (a) Bioreactor setup of *A. manzaensis* grown on ESAE-01 basalt analog material. (b) Box plot of successful recultivation of *A. manzaensis* cells in triplicate sets (A_1–3_ and B_1–3_) after 1 month of desiccation compared to fresh culture (Fc). The median is shown as a black line, and the mean of the samples is indicated by a plus sign (+). Eight data points per sample were obtained, corresponding to cell counts from t_0h_ to t_72h_ (cells/ml).

After the successful cultivation of *A. manzaensis* in culture medium with the presence of Mars analog material ESA01-E (Fig. 3A), a desiccation experiment was conducted on the test samples to verify their stability and resistance to desiccation, which is a critical parameter for Mars-like exposure. The analog material ESA01-E is known for its physicochemical similarity to Martian olivine-rich picrobasalt (Bost et al. 2013, Foucher et al. 2022), and a dehydration experiment was conducted over a period of 1 month. Concentrated cell pellets were deposited into metal exposure wells to investigate cell survival upon the recovery of the dried pellets. The cells were recovered, and cell survival was demonstrated by successful recultivation of previously desiccated cells (Fig. 3B). In the two triplicate sets (A_1–3_ and B_1–3_), cell numbers doubled during the cultivation period of 72 h. The mean number of cells was between 2.5 and 3 × 10^6^ cells/ml throughout the two triplicate sets (A_1–3_ and B_1–3_) compared to a mean number of cells of 4–4.5 × 10^6^ in a fresh culture (Fc). When comparing the triplicate cell counts of the final cell numbers (cells/ml) upon reaching stationary phase at *t* = 72 h, single-factor ANOVA analysis revealed no statistical difference between the two desiccated sets A_1–3_ and B_1–3_ (*P* = .96 with α = 0.05) but statistical differences between Fc, and the two sets taken together A_1–3_ and B_1–3_ (*P* = .01 with α = 0.05), or individually: Fc and A_1–3_ (*P* = .02 with α = 0.05), and Fc and B_1–3_ (*P* = 6.97 × 10–5 with α = 0.05). While the high *P*-value in Fc to B_1–3_ is due to stronger variations within the final cell numbers in B_1–3_, the nonsignificant difference between A_1–3_ and B_1–3_ highlights the comparability of the recultivations, while the significant difference between both Fc and A_1–3_ and Fc and B_1–3_ reflects the effect of desiccation on the cell–mineral mixtures. However, variation in growth was observed in sample A_1_, which could have been caused by a nonhomogeneous mixture of cell–mineral materials used for incubation. Previous dehydration studies performed with *M. sedula* have shown cell revival after two months of desiccation following recultivation, with varying cell numbers based on their mineral substrates, including Mars analog material (Kölbl et al. 2020). This validates their use in subsequent exposure to Mars-like conditions.

### Two-week exposure experiment in a Mars Simulation Facility

**Figure 4:**
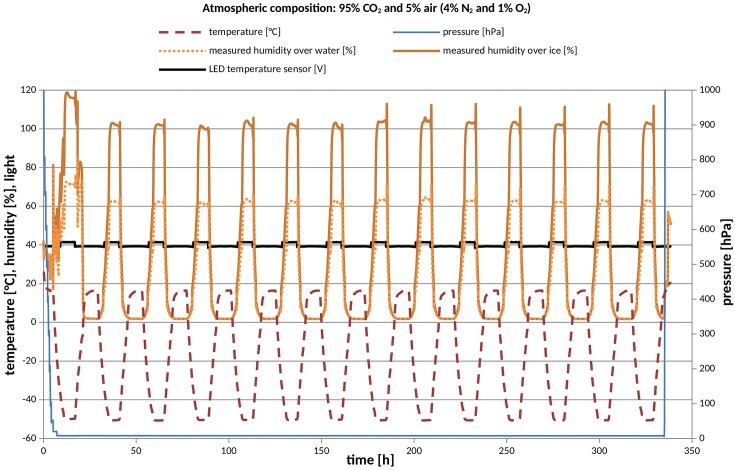
Parameters of the Mars Simulation Facility. The following parameters correspond to an approximate measurement distance of 1 cm above the samples in the sample holder of the Mars Simulation Facility: temperature [°C] and measured humidity over water/ice [%]. The pressure [hPa] gradually decreased and then remained constant. The day/night cycle (16/8 h) was measured using an LED temperature sensor [V].

The sensors integrated in the simulation chamber successfully recorded the physico–chemical parameters of the experiment over the course of 340 h = 14 days, as displayed in Fig. 4. The following parameters correspond to an approximate measurement distance of 1 cm above the samples in the sample holder of the Mars Simulation Facility: temperature [°C], measured humidity over water [%], and measured humidity over ice [%]. Pressure [hPa] is the pressure inside the experimental chamber. The LED temperature sensor [V] represents the day/night cycle (higher values are nighttime and lower values are daytime). All measurements showed excellent adequacy with the input values, successfully recreating day/night cycles.

**Figure 5:**
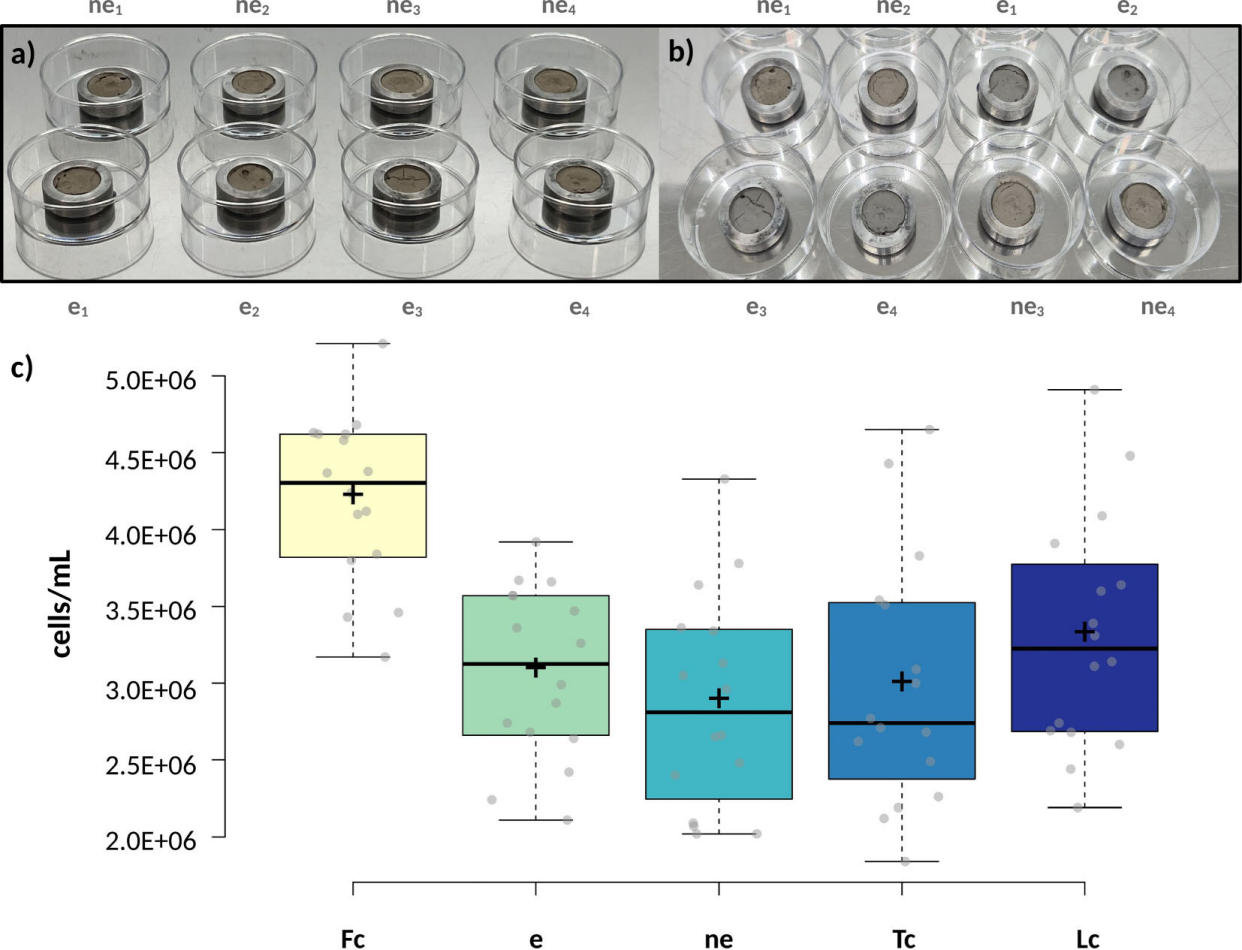
Samples before and after exposure, and recultivation of exposed cells. (a) Metal exposure wells with deposited cell-mineral mixture before exposure: no exposure (ne_1-4_) to UV light; exposure (e_1-4_) to UV light. (b) Metal exposure wells with deposited cell–mineral mixture after 14 days of exposure. (c) Box plot of successful recultivation in triplicate of samples e, ne, Tc, and Lc compared to cell–mineral material recultivated immediately after harvesting (Fc). The median is shown as a black line, and the mean of the samples is indicated by a plus sign (+). Sixteen data points per sample were obtained, corresponding to cell counts from t_0h_ to t_72h_ (cells/ml).

In relation to the samples before exposure (Fig. 5A), a change in the physical appearance (discoloration) of the samples exposed to UV light (e) was observed (Fig. 5B) compared to the unexposed samples (ne). Samples e, ne, Tc, and Lc were successfully recultivated in triplicate upon arrival (Fig. 5C) for 72 h and compared to the recultivation of fresh cell–mineral material (Fc) immediately after harvesting. When comparing the quadruplicate cell counts of the final cell numbers (cells/ml) upon reaching stationary phase at *t* = 72 h, single-factor ANOVA analysis revealed statistical difference between Fc, e, ne, Lc, and Tc (*P* = .01 with α = 0.05) and between Fc, e, and ne (*P* = 3.91 × 10–5 with α = 0.05) but no statistical difference between the remaining samples. This highlights the difference between the fresh culture (Fc) and the prepared samples for the exposure experiment e, ne, Tc, and Lc, as well as the strong variation between Fc, e, and ne. Although Fc showed the highest initial cell number, no significant trend was observed between e, ne, Tc, and Lc. However, the cell number increased in comparison to initial concentration by 1 × 10^6^ cells/ml in all samples.

No significant difference was noted after 1 month of desiccation in normal atmosphere and 8 hPa for 2 weeks. Although UV-exposed samples appeared bleached in appearance, the survival fraction was not affected by radiation exposure, with very comparable values reached after recultivation across all sets. These results are in agreement with those of previous experiments conducted by Mastascusa et al. (2014), who assessed the survival by successful recultivation of the comparable extremely thermoacidophilic archaea *Sulfolobus solfataricus*, in the same order as *A. manzaensis* (Sulfolobales), to simulated space conditions: pronounced resilience to thermal fluctuations (1 week of −196°C to 85°C) and a high resistance to UV exposure (1 h to λ = 254 nm). However, regarding UV exposure, it must be considered that in our experiments, we did not expose thin films spread in Petri dishes, but cell–mineral mixtures in metal exposure wells. Therefore, only the first millimeter was irradiated, and the fraction below the surface largely contributed to the survival, which was subjected to exposure to other extreme conditions, such as temperature variations, pressure, and humidity. No precise measurements were taken regarding the specific penetration depth of UVs in the tested samples; however, earlier studies suggest that a few millimeters of regolith are sufficient to significantly block harmful UV radiation (shorter than 300 nm) (Carrier et al. 2019).

### Astrobiological relevance of thiophene-bearing quinones

**Figure 6:**
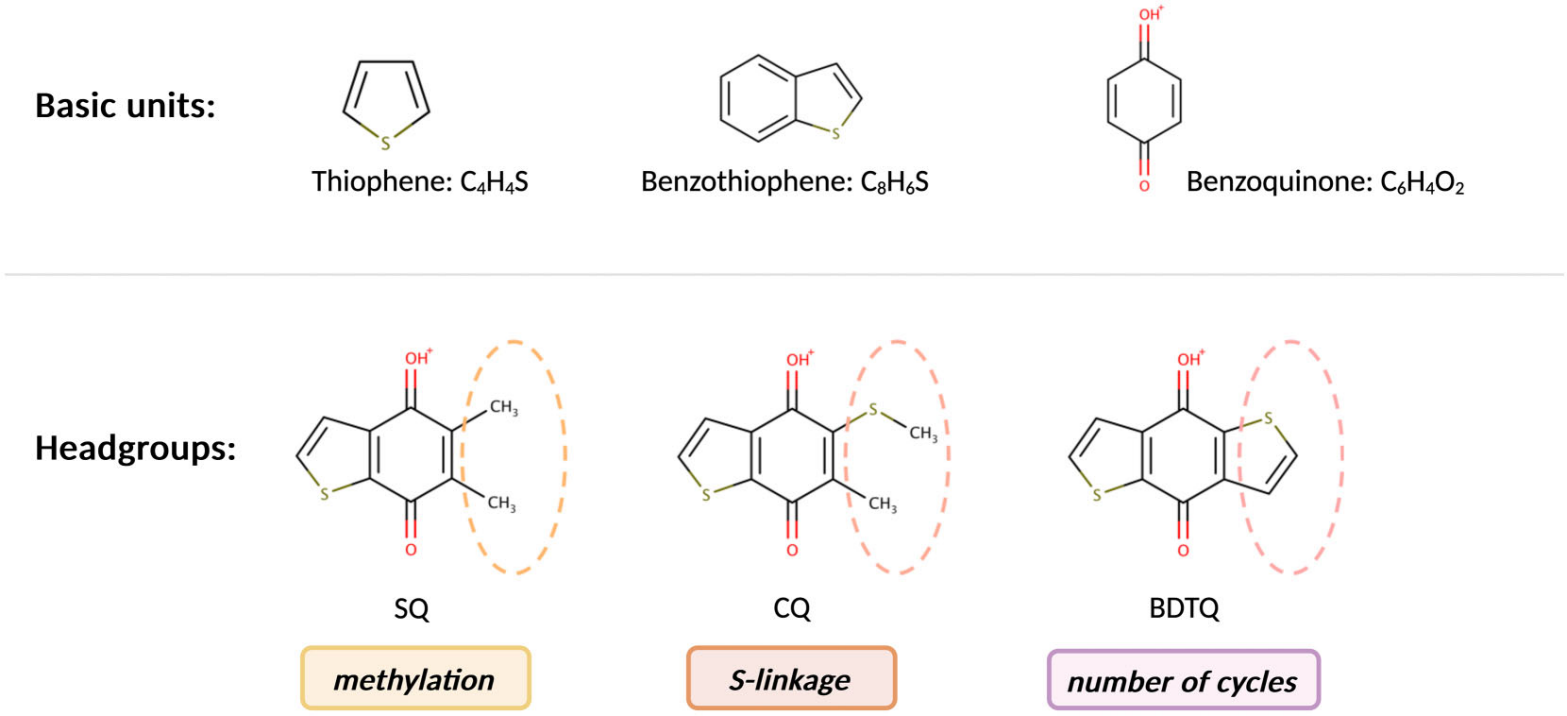
Main structural features of thiophene-bearing quinones. The headgroups of sulfolobusquinones (SQ), caldariellaquinones (CQ), benzodithiophenequinones (BDTQ), and their basic units consist of thiophenes (C_4_H_4_S), benzothiophenes (C_8_H_6_S), and benzoquinone (C_6_H_4_O_2_). A comparison of headgroup characteristics revealed differences in methylation, S-linkage, and/or number of cycles.

The detection of thiophenes on Mars (Eigenbrode et al. 2018) has initiated an ongoing discussion regarding their potential biogenic origin (Heinz and Schulze-Makuch 2020). Therefore, we examined the thiophene-bearing quinone headgroups of Sulfolobales and their basic units. A closer examination of the differences in the thiophene headgroup properties (Fig. 6) revealed potential molecular target sites for molecular alterations in the form of methylation, S-linkage properties, and/or the number of cycles. However, exposure to Mars-like conditions may alter molecular signatures over time. Similar potential biosignificant target sites for molecular alterations have been reported for lipid biomarkers (Finkel et al. 2025), including unsaturation, methyl groups, and/or polycyclic ring structures, identifying saturated chains with functional groups as the “most reliable biomarkers for long-term preservation,” especially when embedded and thus shielded from radiation in appropriate mineral matrices (Finkel et al. 2023). Therefore, using cell–mineral mixtures and after an initial desiccation experiment to establish the necessary methodology for molecular analysis and recultivation techniques, an exposure experiment under Mars-like conditions was conducted to test not only the resilience of the microbial cells to Mars-like conditions but also to further elucidate the role of these molecules as potential biosignatures for life on Mars and other celestial bodies.

Planetary environmental conditions must be considered when assessing the preservation potential of biomarkers over the course of billions of years. On Earth, the occurrence of thiophenes associated with Archaean sediments (4.0–2.5 Ga) is related to the diagenetic and metamorphic histories of the sediments (French et al. 2015). Primary organic matter in microbial phototrophic colonies is degraded by heterotrophic microbes, such as sulfur reducers, during which the sulfur content of the organic matter is enriched (Westall et al. 2011). Metamorphism converts sulfur-containing molecules into aromatic organic structures, such as thiophenes. However, Mars’ metamorphic history is different from that of Earth. The planet is approximately half the size of Earth and lacks plate tectonics. Therefore, any sedimentary rock would have been subjected to a lower degree of metamorphism, except for shock metamorphism from meteorite impacts (Osinski et al. 2020, Bosak et al. 2021). Therefore, it is important to consider the general environmental context and geological setting to distinguish between plausible biotic and abiotic settings. Exposure of thiophene-bearing quinones at the surface of Mars to 3–4 Ga of UV, galactic, and cosmic radiation will affect the survivability of the basic thiophene moieties; however, data from Gale Crater on Mars (Eigenbrode et al. 2018) show that they may still be preserved in the Martian environment, although their biogenicity remains to be proven.

### Thiophene-bearing quinones after exposure to Mars-like conditions

**Figure 7:**
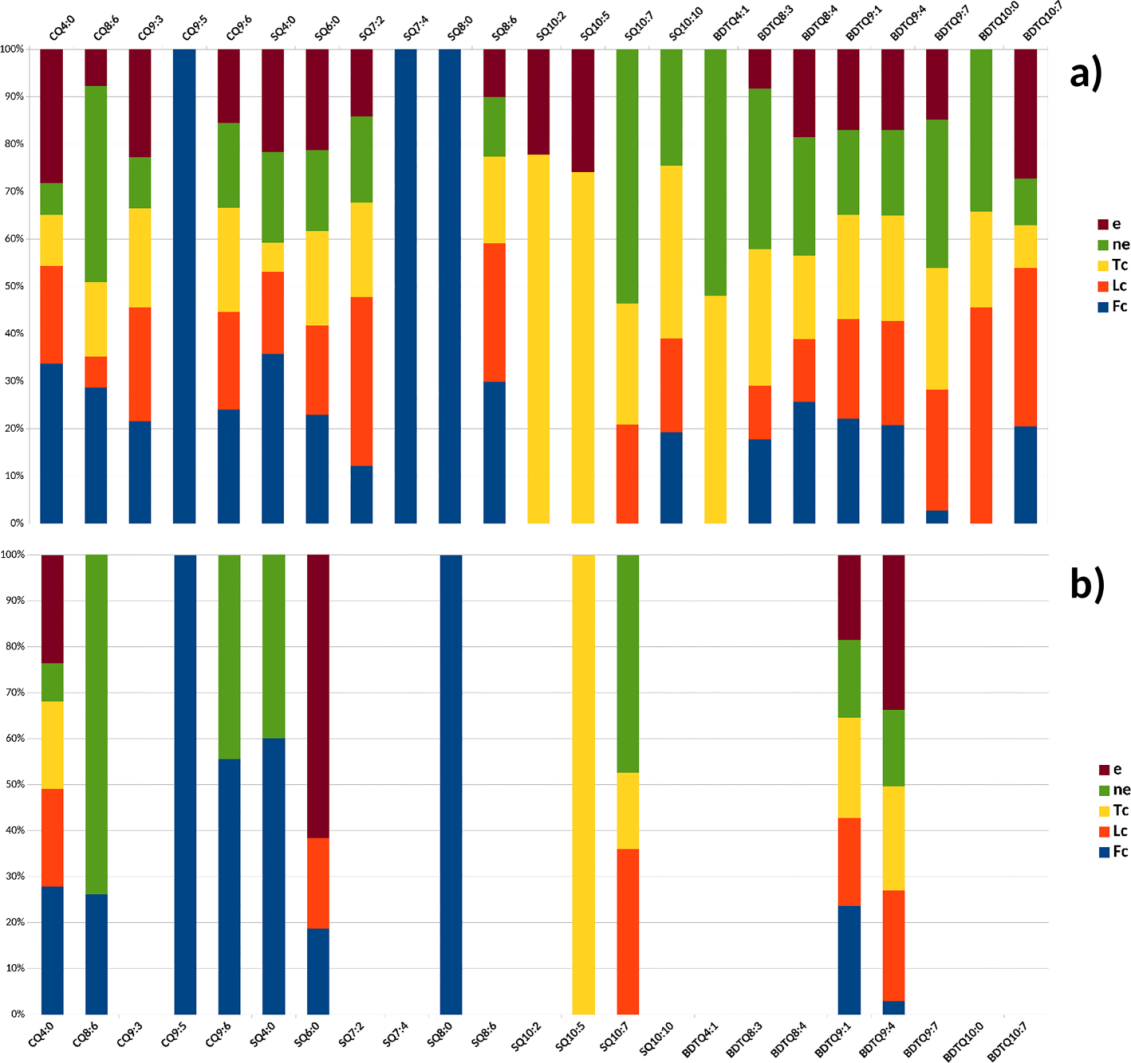
Tentative annotation of thiophene-bearing quinones. (a) Summed peak areas of putatively annotated thiophen-bearing quinones (CQ_n: x_, SQ_n: x_, and BDTQ_n: x_) in the MS spectra: exposed (e), nonexposed (ne), transport control (Tc), laboratory control (Lc), and fresh culture (Fc). (b) Summed peak areas of the subset of tentatively annotated thiophen-bearing quinones, including only features detected in measurements for which supporting MS/MS spectra were available. See Fig. S1 for a reversed axis representation.

The first tentative annotation based on MS analysis using MetaboScape® (Table S1) across all samples (e, ne, Tc, Lc, and Fc) revealed the heterogeneous distribution of differently oxidized thiophene-bearing quinones (Fig. 7A). We were able to annotate for the following oxidation states for CQs: CQ_4:0_, CQ_8:6_, CQ_9:3_, CQ_9:5_, CQ_9:6_; for SQs: SQ_4:0_, SQ_6:0_, SQ_7:2_, SQ_7:4_, SQ_8:0_, SQ_8:6_, SQ_10:2_, SQ_10:5_, SQ_10:7_, SQ_10:10_; and for BDTQs: BDTQ_4:1_, BDTQ_8:3_, BDTQ_8:4_, BDTQ_9:1_, BDTQ_9:4_, BDTQ_9:7_, BDTQ_10:0_, BDTQ_10:7_.

The SQ_10_ columns showed a drop in the number of double bonds with exposure: a higher proportion of double bond SQ_10_ (SQ_10:10_ and SQ_10:7_) was found in samples that were not exposed compared to exposed samples (which only had SQ_10_ with a lower number of double bonds). This could be the result of UV radiation breaking these bonds. However, while the laboratory control (Lc) did not exhibit a drop in the proportion of double bonds, the control sample (Fc) did not exhibit these characteristics. Certain indicative features, CQ_9:5_, SQ_7:4_, and SQ_8:0_, were only found in the samples extracted immediately after cultivation. SQ_10:2_ and SQ_10:5_ were present in the exposed samples and the transport control, whereas SQ_10:7_, SQ_10:10_, BDTQ_4:1_, and BDTQ_10:0_ were absent in the exposed samples. This poses a challenge when ascribing features to actual exposure. The analysed samples were organic extracts of homogenized material immediately analysed after exposure to resolve minor changes in the thiophene-bearing quinone headgroups visible by energetic fragmentation using MS/MS. However, the generally low yield of the corresponding MS/MS spectra (Fig. 7B) complicates this approach, requiring further optimization. As a consequence of the low yield of confirmatory MS/MS spectra, the aforementioned feature annotation should be considered tentative, and future experiments should aim to prioritize additional MS/MS acquisition to further validate the proposed structures and increase annotation confidence. Because of the low penetration depth of UV radiation in the cell–mineral mixture in the metal exposure wells, only a small fraction of the cells was exposed to the degradation of thiophene-bearing quinones, particularly their thiophene moieties. A different sample preparation procedure might also solve this problem; however, complementary analyses, such as survival assays, would not have been possible if thin films were used in Petri dishes. The purification of thiophene-bearing quinones and their direct exposure to UV radiation are other alternatives. A more powerful radiation source may better mirror the induced changes in the geological timescales of thiophene moieties and ultimately shed light on specific remnant molecular markers of biologically produced thiophenes. Additionally, artificial fossilization and diagenesis of these compounds would allow for a stepwise reconstruction of molecular alterations, which could be compared with organic remnants in the geological record and direct data available from current and future Mars missions. Therefore, we propose the continuation of an irradiation experiment with a proton ion source (e.g. Cyclotron at the CEMHTI laboratory in Orléans, France) or an exposure platform in a Low Earth Orbit, as shown by the completed BIOMEX (de Vera et al. 2019) and planned BioSigN projects (de Vera 2019) or future planned space experiment platforms (Elsaesser et al. 2023).

## Conclusion

We successfully recultivated *A. manzaensis* cells after exposure to 1 month of desiccation, demonstrating their survival under water depletion and two weeks of simulated Mars-like conditions, proving their survival under extreme temperature fluctuations ranging from +15 to −50°C in diurnal cycles (16 h daylight and 8 h night), exposure to UV radiation (200–2200 nm), Martian atmosphere (95% CO_2_ and 5% air composed of 4% N_2_ and 1% O_2_), and pressure (8 hPa). This highlights the relevance of thermophilic archaea in astrobiology research, particularly *A. manzaensis*, as a model organism. Improved sample preparation and different radiation sources are being evaluated to further assess the relevance of thiophene-bearing quinones as model biosignatures, which will guide current and future missions in the search for signs of life on Mars.

## Supplementary Material

xtag043_Supplemental_Files

## Data Availability

The data underlying this article are available in the article and its online supplementary material.
