# Supplementary material for: Assessing cell survival and biosignature preservation of the extremely thermoacidophilic archaeon Acidianus manzaensis after exposure to Mars-like conditions
Source: FEMS Microbes. 2026 Jul 30;7:xtag043. doi: 10.1093/femsmc/xtag043 (PMC13471030; doi:10.1093/femsmc/xtag043)
Supplement: Supplementary file 1 [file xtag043_supplemental_files.zip › FEMSMC-2025-082.R1 supplementary data - reviews and author response.docx]

**Reviews of manuscript FEMSMC-2025-082**

**Reviewer 1: Mara Leite**

**Recommendation: Minor Revision**

The manuscript is well-written, logically structured, and accessible. The introduction provides a comprehensive foundation, while the goals and methodology are clearly articulated and relevant to current trends in astrobiology and planetary science. Moreover, the authors correctly identify that the low penetration depth of UV radiation in the cell-mineral mixture limits the fraction of exposed biomass. To enhance the technical precision and readability of the manuscript, I suggest the minor adjustments in the attached file.

Attached file:

By integrating extreme temperature variations, desiccation, and radiation, the authors effectively address the three primary environmental "killers" on the Martian surface. Furthermore, the decision to conduct these experiments "in the presence of mineral materials" (using the ESA01-E analog) is a significant strength; it elevates the study from a standard laboratory culture experiment to a high-fidelity simulation of a realistic Martian soil environment. The dual focus on both microbial survival and biosignature preservation provides a holistic view of habitability that is essential for future life-detection missions.

To enhance the technical precision and readability of the manuscript, I suggest the following minor adjustments to the language:

-	Line 44: This is a good line to strengthen the Paleo-Mars Connection. I suggest mentioning that these microbes serve as excellent analogs for early Mars due to their capacity to tolerate the intense volcanic heat and acidic gases prevalent during that era. While volcanism is mentioned earlier, this is the ideal location to solidify the link between the organism's physiology and the geochemically active Martian past.

-	Line 104: Even though methalogoy is presented in detail I think this is a good line to clarify growth strategy. The manuscript mentions that A. manzaensis is an autotroph, yet a heterotrophic medium is used for recovery. While using organic supplements (like yeast extract) is a common and effective technique to "jumpstart" the recovery of stressed cells, the authors should specify here whether the medium was merely supplemented or if the cells were shifted entirely to heterotrophic growth. This is a critical detail, as metabolic shifts can significantly alter the resulting isoprenoid quinone profiles.

-	Line 157: The description of air-drying under a laminar flow hood and wrapping samples in aluminum foil is an excellent methodological detail. However, the authors should explicitly state why this is critical: many lipids and quinones are highly photosensitive and shielding from ambient light is therefore essential to avoid non-Martian biomarker degradation.

-	Line 195: In the description of visual cell counting, I recommend replacing the term "relief" with more technically precise terminology such as "optical properties" or "refractive index." This better describes the visual distinction between the spherical microbial cells and the irregular mineral particles under phase-contrast or light microscopy.

-	Line 287: The phrase "good resistance" to temperature variations should be replaced with a more robust scientific descriptor. Consider using "robust thermal tolerance" or "pronounced resilience to thermal fluctuations." These terms are more scientifically appropriate.

**Reviewer 2**

**Recommendation: Major Revision**

It is an interesting article, a nice experiment and appropriate for the current research lines in astrobiology and biosignature detection, however data analysis needs to looked at again (or requires clarification as to why certain analysis were done). Results are still relevant, so points made in the revision about the experimental plan are mainly for future experiments and reproducibility issues.

Full Review:

Review: Assessing cell survival and biosignature preservation of the extremely thermoacidophilic archaeon Acidianus manzaensis after exposure to Mars-like conditions

The article is well written, starts really strong and has and interesting experiment, however the discussion and data analysis seem to have been cut short.

I have a few questions about the methodologies and I think there is room for improvement regarding the mass spec results. Certain decisions regarding data analysis and the experimental plan are not clear without more clarifying descriptions of the statistical analyses chosen.

I think this article contains data that merits publication but a revision of the discussion is needed. For future experiments, certain conditions might need to be tested independently to get a better understanding of this organisms and the effects that the martian environment has on its biosignatures.

Line 58: Regarding the quinone structure: is there anything that can be said specifically about the differences between abiotic and biotic fossils? In the discussion a diagram of the different head groups is shown, and the discussion itself relates to degradation or these organics, etc. So, it would be interesting to see if the degradation results in the same headgroup, differences in double bonds etc. Either a diagram (can be from other papers if referenced) or a sentence describing an example.

Line 81, 86 and 87: For the first mention of these organisms in the article, don’t abbreviate the genus (there is a none-abbreviated later on in line 286).

Line 90: You jump from the description of quinones and its proportion to the survival of A. manzaensis. Many of the references in line 76

Line 98: The experimental plan and results are centered around dessication and dessication+radiation. Temperature is not accounted for as a separate experimental control so its effects on the degradation of organics and stress on A. manzaensis, is hard to infer. Basically, there is an issue with the degrees of freedom in the experiment. The effect of temperature, pressure, water activity, oxygen, etc cannot be isolated from one another and the dessication+radiation, in which case you would need more controls to account for it. I would reword this sentence, moving the word “temperature” and maybe stating: “In this study, we focused on the effects of extreme desiccation and radiation on the survival of A. manzaensis formation of potential biosignatures in the presence of mineral materials and under martian conditions (temperature and pressure).”

Line 122: Minor rewording thing: When you say “re-cultivated together” I’m guessing you are saying “at the same time” but the wording here makes it seem samples from different experiment were grown in the bioreactor together.

Figure 1: Recultivation of the exposed cells to determine cell survival shows a picture of the bioreactor again. I would add the time it took to grow the cells again (which I imagine was 96h too).

Line 148: Was the microbe cultivated in media and martian basalt or just water and martian basalt? If it is under anaerobic like in the protocol cited I would add “anaerobically” when mentioning the growth conditions as the presence of oxygen has a major effect on the way samples need to be handled, how it affects organics, etc. If the researchers took any measure to avoid oxygen contamination (transferring samples, dessicating, etc) it should be mentioned more often.

Line 150: I would add if the cell culture was concentrated through centrifugation and at what speed and for how long. When “homogenized” was the supernatant removed and 40 mL left to resuspend the cells or was this fresh media + basalt. If no fresh liquid is used, the process of concentrating the cells will also concentrate the ratio of basalt to media (or water), effectively increasing the salinity.

Line 167: Mention temperature and humidity values.

Figure 2: The platform inside the chamber is said to rotate but I’m guessing it wasn’t necessary as half of the samples are always in the dark part. I would remove the word rotating in the figure and text or specify if it was used or not. First glance at figure 2 I thought the samples were rotating to simulated day/night cycles instead of turning the UV light on and off.

Line 179: Was each half weighed? I understand that it won’t matter much for the molecular analysis of the quinones but it could affect the reproducibility of experiments looking at cell number, absolute organic concentrations, etc.

Line 186: 1 mL concentrated cell-mineral material. Same issue as the homogonized samples mentioned in Line 150.

Line 187: This is an anaerobically grown microbe. I would mention it here as handling of samples is very different.

Line 188: Yoshida et al. 2006 already contains trace elements. Is the Allen trace element solution used as a replacement or in addition?

Line 189: Tryptone is mentioned which I understand is used as an electron donor, but Fe2O3 is not a suitable electron acceptor for this strain.

Line 232: I would again clarify here if this culturing step was done with just the analogue material or if it was also supplemented with media. If it is the later, I would change the wording to “successful cultivation in the presence of analog material…”

Figure 3: I would add one column for a growth control if you have the data (or even if there is data online). A cell count for a sample not exposed or dessicated, just subcultured, as a comparison or even the Fc set which feels like is the baseline control for every other condition.

Line 260: Consider copying or moving these values to the methods section (line 167).

Line 281: Is there no significant difference between the two triplicate sets of desiccated samples, or the desiccated samples when compared to a recultivated, non-exposed sample (e.g. Fc)? Also, if you did some kind of significance test, I would add the significance value here and consider adding a line or two about data analysis/statistics, with the type of test used, in the methods, not just the specific package or online tool.

Figure 7: This figure is ok, but I wonder if the authors could change it for, or add a version (maybe move one to the supplemental material) where the groups are switched: samples in the X axis and quinone proportions in the legend to help visualize differences between samples instead of differences between proportions of quinones. The main aim is not to profile quinones in A. manzaensis (that is secondary) but measure the effects of the UV (that is your independent variable in this case).

Line 340: do you have a value for the penetration of UV and the thickness of the samples? If the discoloration described previously affected the entire sample, I would assume the UV did penetrate the sample, unless measured.

Line 343: it is not clear why other sample preparation procedures, like the “thin films” mentioned wouldn’t be effective. Is the volume a problem, e.g not enough cells for organic extractions?

Discussion: The discussion starts with a description of potential targets for biosignature detection and the differences between these molecules but the discussion of the results in figure 7 make little to no mention of the differences found, therefore the section 295-301, might need slight rewording. Not familiar with these organics, but I feel like much more can be said from the mass spec results, including statistical analysis between the proportion of different types of quinones for each sample type, and not just the presence/absence of quinones (I’ve made a point below about absolute values too). For example, SQ10 columns show a drop in the number of double bonds with exposure: higher proportion of double bond SQ10 (SQ10:10 and SQ10:7) are found in samples that were not exposed compared to exposed samples (which only had SQ10 with lower number of double bonds). This could be the result of the UV breaking these bonds. Even if the control sample (Fc) lacks these, I think it is still valuable information.

Are the proportions of total extracted organic compounds, cell numbers or sample weight used to normalize the proportion of quinones between samples? I couldn’t find how this was done, and it could affect the proportions in figure 7, e.g. sample Fc having a higher organic yield could affect the result if it is not considered. The article referenced in the methods only deals with one sample. Maybe I’ve missed something. I understand that mass spec data is not always useful for quantifying absolute concentrations of certain organics but then it should be mentioned in the text, either the methods or the discussion. When the data is presented this way it is inferred that the proportions of organics is the result form the data. If the data is not significant enough or the authors wanted to focus just in absence/presence then it should be mentioned in the methodology and a different graph considered, such as a heatmap or a table.

If you are considering carrying on with these experiments in the future, survival assays could be done with replicating stamps (to recover only the top layer), or protein/ATP assays, qPCR, transcriptomics, live/dead stains, etc. Part of the sample was divided using a sterile scalpel so I’m guessing the number of runs or access to the martian reactor was limited but it might be useful to either subdivide the sample holders themselves or use the same scalpel to separate top/bottom layers, even if just to see differences in cell counts. I think the discussion about why the UV exposure wasn’t effective should be expanded with one or two sentences and maybe a reference. I think the article needs to clarify whether figuring out if shielding of the organics by the martian basalt is the main aim of the experiments or if it is the stress on cells yields different proportions of organics. The introduction makes it seem it is the former but the experimental plan is much more similar to the later. The authors discussed a future experiment with just the organics which make me lean towards thinking the aim was to see the response on the cells, in which case, growing them with martian regolith is appropriate but then the cells could have been washed (e.g. through centrifugation and fresh media or PBS) or the basalt decanted to focus on the effect of UV on the cells. I think there needs to be on of the two experiments before this one to get a better understanding of what is happening.

I would like to see a mention about oxygen in the discussion or the methods. The organism selected is grown anaerobically following the protocol from Yoshida *et al.* 2006 but air desiccated. The introduction also mentions how the presence of oxygen during growth of other species affects the proportion of quinones. I would include what considerations were undertaken to deal with oxygen contamination or how it was handled a posteriori statistically. In the future there might need to be an oxygen/no oxygen control. Similarly controls could have been carried out for isolating and measuring the effects of temperature.

**Associate Editor Recommendation: Major Revision**

Dear Authors,

Thank you for submitting to our special collection and for your patience with the review process. Whilst your article is of great astrobiology interest and it would be a great addition to our collection, important considerations were highlighted in the review process. Addressing those would make the article even stronger and we would be very happy to have it in the collection.

**EIC Decision: Major Revision**

**Author's Response to Decision Letter for (FEMSMC-2025-082)**

Dear editorial board of FEMS Microbes,

Dear Reviewer 1 and Reviewer 2,

First, we would like to thank the reviewers for their reports, which allowed us to improve the overall quality of our paper. We are grateful for your constructive comments and the opportunity to resubmit a new version of our manuscript. We have adapted this new version of the manuscript according to your guidelines. Please refer to the answers to the individual comments below. The reviewers’ comments are in black and our answers are in green, while the lines are marked as Line XYZ.

Sincerely,

Sebastian Gfellner on behalf of myself and my co-authors

**_____________________________________________________________________Reviewer 1**

By integrating extreme temperature variations, desiccation, and radiation, the authors effectively address the three primary environmental "killers" on the Martian surface. Furthermore, the decision to conduct these experiments "in the presence of mineral materials" (using the ESA01-E analog) is a significant strength; it elevates the study from a standard laboratory culture experiment to a high-fidelity simulation of a realistic Martian soil environment. The dual focus on both microbial survival and biosignature preservation provides a holistic view of habitability that is essential for future life-detection missions.

To enhance the technical precision and readability of the manuscript, I suggest the following minor adjustments to the language:

**Line 44:** This is a good line to strengthen the Paleo-Mars Connection. I suggest mentioning that these microbes serve as excellent analogs for early Mars due to their capacity to tolerate the intense volcanic heat and acidic gases prevalent during that era. While volcanism is mentioned earlier, this is the ideal location to solidify the link between the organism's physiology and the geochemically active Martian past.

Thank you for the comment; we have added the following text in Line 44: “In particular, these microorganisms serve as excellent analogs for early Mars because of their physiology and capacity to tolerate the intense volcanic heat and acidic gases that were prevalent during the geochemically active Martian past (Lewis *et al.* 2018; 2021).”

**Line 104:** Even though methodology is presented in detail I think this is a good line to clarify growth strategy. The manuscript mentions that A. manzaensis is an autotroph, yet a heterotrophic medium is used for recovery. While using organic supplements (like yeast extract) is a common and effective technique to "jumpstart" the recovery of stressed cells, the authors should specify here whether the medium was merely supplemented or if the cells were shifted entirely to heterotrophic growth. This is a critical detail, as metabolic shifts can significantly alter the resulting isoprenoid quinone profiles.

Thank you for your comment and for pointing out this important aspect. No heterotrophic medium was used in this study. Only tryptone was used as the N-source, and no additional organic supplements were added. In this process, concentrated cell pellets of *A. manzaensis* were deposited into metal exposure wells to monitor cell survival upon recovery of the dried pellets in a strain-specific autotrophic medium supplemented with tryptone as a nitrogen source (Yoshida et al., 2006).

**Line 157:** The description of air-drying under a laminar flow hood and wrapping samples in aluminum foil is an excellent methodological detail. However, the authors should explicitly state why this is critical: many lipids and quinones are highly photosensitive and shielding from ambient light is therefore essential to avoid non-Martian biomarker degradation.

Thank you for the comment; the following explanation has been added in Line 193: “The samples were wrapped in sterilized aluminum foil, which is critical because many lipids and quinones are highly photosensitive, and shielding them from ambient light is essential to avoid biomarker degradation during storage.”

**Line 195:** In the description of visual cell counting, I recommend replacing the term "relief" with more technically precise terminology such as "optical properties" or "refractive index." This better describes the visual distinction between the spherical microbial cells and the irregular mineral particles under phase-contrast or light microscopy.

Thank you for the comment; “refractive index” has been added.

**Line 287:** The phrase "good resistance" to temperature variations should be replaced with a more robust scientific descriptor. Consider using "robust thermal tolerance" or "pronounced resilience to thermal fluctuations." These terms are more scientifically appropriate.

Thank you for the comment; “pronounced resilience to thermal fluctuations” has been added.

**_____________________________________________________________________Reviewer 2**

Review: Assessing cell survival and biosignature preservation of the extremely thermoacidophilic archaeon Acidianus manzaensis after exposure to Mars-like conditions

The article is well written, starts really strong and has and interesting experiment, however the discussion and data analysis seem to have been cut short.

I have a few questions about the methodologies and I think there is room for improvement regarding the mass spec results. Certain decisions regarding data analysis and the experimental plan are not clear without more clarifying descriptions of the statistical analyses chosen.

I think this article contains data that merits publication but a revision of the discussion is needed. For future experiments, certain conditions might need to be tested independently to get a better understanding of this organisms and the effects that the martian environment has on its biosignatures.

**Line 58:** Regarding the quinone structure: is there anything that can be said specifically about the differences between abiotic and biotic fossils? In the discussion a diagram of the different head groups is shown, and the discussion itself relates to degradation or these organics, etc. So, it would be interesting to see if the degradation results in the same headgroup, differences in double bonds etc. Either a diagram (can be from other papers if referenced) or a sentence describing an example.

Thank you for your comment. There is little literature on direct comparisons of quinones in abiotic versus biotic fossils. Thus, we are fostering a discussion on changing this. In particular, early findings of thiophenes in the rock record (2.72 Ga) were reported by Lepot *et al.* (2009), ascribing a high amount of organic sulfur in correlation with shifts in C=C bonds similar to the energy of thiophene groups based on EDXS spectra. Therefore, the next steps for future experiments would be to artificially fossilize thiophene-bearing quinones to answer this question. We added a corresponding outlook sentence in Line 432: “Additionally, artificial fossilization and diagenesis of these compounds would allow for a stepwise reconstruction of molecular alterations, which could be compared with organic remnants in the geological record and direct data available from current and future Mars missions.”

**Line 81, 86 and 87:** For the first mention of these organisms in the article, don’t abbreviate the genus (there is a none-abbreviated later on in line 286).

Thank you for spotting this oversight; it has been corrected.

**Line 90:** You jump from the description of quinones and its proportion to the survival of A. manzaensis. Many of the references in line 76

Thank you for your comment. We improved the transition and added the following sentences between the description of quinones and its proportion to the survival of *A. manzaensis* in Line 94: “However, quinone distribution may vary under extreme environmental conditions. In particular, the harsh conditions of the Martian surface must be considered when examining not only the potential survival of microorganisms but also the preservation of these compounds.”

**Line 98:** The experimental plan and results are centered around dessication and dessication+radiation. Temperature is not accounted for as a separate experimental control so its effects on the degradation of organics and stress on A. manzaensis, is hard to infer. Basically, there is an issue with the degrees of freedom in the experiment. The effect of temperature, pressure, water activity, oxygen, etc cannot be isolated from one another and the dessication+radiation, in which case you would need more controls to account for it. I would reword this sentence, moving the word “temperature” and maybe stating: “In this study, we focused on the effects of extreme desiccation and radiation on the survival of A. manzaensis formation of potential biosignatures in the presence of mineral materials and under martian conditions (temperature and pressure).”

Thank you for your comment. Although single parameters have been explored to ensure an optimal scientific output of the complete Mars simulation, we focused on the main damaging factors of desiccation and radiation. The sentence has been reworded as suggested to better separate the different stressors and the implied results.

**Line 122:** Minor rewording thing: When you say “re-cultivated together” I’m guessing you are saying “at the same time” but the wording here makes it seem samples from different experiment were grown in the bioreactor together.

Thank you for the comment; the sentence has been rewritten as suggested.

**Figure 1:** Recultivation of the exposed cells to determine cell survival shows a picture of the bioreactor again. I would add the time it took to grow the cells again (which I imagine was 96h too).

Thank you for the comment; the final growth time has been added as suggested.

Line 148: Was the microbe cultivated in media and martian basalt or just water and martian basalt? If it is under anaerobic like in the protocol cited I would add “anaerobically” when mentioning the growth conditions as the presence of oxygen has a major effect on the way samples need to be handled, how it affects organics, etc. If the researchers took any measure to avoid oxygen contamination (transferring samples, desiccating, etc) it should be mentioned more often.

Thank you for the question; it has been clarified in the text in Line 160 as follow: “*A. manzaensis* (NBRC 100595) was cultivated aerobically in a 1 L glassblower modified Schott-bottle bioreactor (Duran DWK Life Sciences GmbH, Wertheim/Main, Germany) with culture medium and Mars analog material (ESA01-E basalt, 10 g/L).” Furthermore, the conditions for microbial cultivation are specified in more detail in the following section.

**Line 150:** I would add if the cell culture was concentrated through centrifugation and at what speed and for how long. When “homogenized” was the supernatant removed and 40 mL left to resuspend the cells or was this fresh media + basalt. If no fresh liquid is used, the process of concentrating the cells will also concentrate the ratio of basalt to media (or water), effectively increasing the salinity.

Thank you for the comment. The centrifugation speed and time were added. The supernatant was continuously removed, and no fresh liquid was added. This has been clarified in the manuscript in Line 178 as follow: “To monitor microbial growth, the culture was sampled continuously during the growth phase, and the cells were counted visually under a microscope (Olympus BX51 equipped with a Pixelink M20C-CYL camera) using a Neubauer Chamber (Carl Roth GmbH & Co. KG, Karlsruhe, Germany) and harvested upon reaching the stationary phase. Harvesting was performed by centrifugation in sterile 50 mL Falcon tubes at 3220 × g for 40 min. After harvesting, 800 mL of cell-mineral material was concentrated by a factor of 20 through centrifugation (3220 × g for 40 min). The supernatant was continuously removed, and the cell-mineral material was homogenized in a sterile 50 mL Falcon tube with additional fresh medium to a total volume of 40 mL, and stored at 4°C for 72h before deposition in the metal exposure wells. The deposition was performed by pipetting 300 µL into each metal exposure well and continuously evaporating to a total volume of 2 mL. The samples were stored at room temperature before and during transportation to and from the exposure facility.”

**Line 167:** Mention temperature and humidity values.

Thank you for the suggestion; the temperature and humidity values have been added: “(day-to-night: temperature of +15 to -50°C and relative humidity of 0 to 75%)”

**Figure 2:** The platform inside the chamber is said to rotate but I’m guessing it wasn’t necessary as half of the samples are always in the dark part. I would remove the word rotating in the figure and text or specify if it was used or not. First glance at figure 2 I thought the samples were rotating to simulated day/night cycles instead of turning the UV light on and off.

Thank you for the comment. Indeed, the rotating platform was not used in this experiment, as it was for others, using an in situ photosynthetic sensor (Mini-PAM) to measure fluorescence values. We added the following clarification: “However, for the conducted experiment, the platform was not rotated, but the UV light at the illuminated side was turned on and off to simulate the day/night cycle.”

**Line 179:** Was each half weighed? I understand that it won’t matter much for the molecular analysis of the quinones but it could affect the reproducibility of experiments looking at cell number, absolute organic concentrations, etc.

Thank you for the question. Unfortunately, each half was not weighted, as the fragility of the cell material allowed only direct transfer into the re-cultivation funnel and further extraction. However, the cell/mL count accounts for the reproducibility of the experiment. As the reviewer stated, the molecular analysis was not affected and was normalized by comparing the cell counts.

**Line 186:** 1 mL concentrated cell-mineral material. Same issue as the homogenized samples mentioned in Line 150.

Thank you for your comment. The homogenizing step has been specified in Line 183: “The supernatant was continuously removed, and the cell-mineral material was homogenized in a sterile 50 mL Falcon tube with additional fresh medium to a total volume of 40 mL, and stored at 4°C for 72h before deposition in the metal exposure wells.”, and the following was rewritten in Line 231: “Cultivation was conducted in a shake incubator set at 75°C and 100 rpm over a period of 72h in 50 mL glass flasks containing an equivalent of 1 mL concentrated cell-mineral material and 20 mL of *A. manzaensis* specific medium, including ESA01-E basalt and tryptone, as previously described.”

**Line 187:** This is an anaerobically grown microbe. I would mention it here as handling of samples is very different.

Thank you for your comment. The microbes have been grown aerobically under a constant stream of CO_2_ as has been added in the cultivation in Line 176: “The bioreactor was maintained at a constant temperature of 73°C with steady stirring, and a continuous flow of CO_2_ at a total rate of 0.3 L/min (normalized to 1 atm and 0°C) was ensured.”

**Line 188:** Yoshida et al. 2006 already contains trace elements. Is the Allen trace element solution used as a replacement or in addition?

Thank you for your comment. Allen trace element solution was used as a replacement. We followed the A. manzaensis medium as described by Kölbl et al. (2022) with tryptone as N-source, as described by Yoshida et al. (2006). The trace element solution corresponds to the trace elements provided by Yoshida et al. (2006) medium, which was modified by Kölbl et al. (2022). As our recultivation experiments were aligned with those of Kölbl et al. (2020) and Kölbl et al. (2022), we adapted their methodology for the cultivation medium and desiccation experiments. We have specified the culture medium used in Line 167: “Cultivation was conducted using *A. manzaensis* specific medium consisting of 0.132 g/L (NH_4_)_2_SO_4_, 0.041 g/L KH_2_PO_4_, 0.49 g/L MgSO_4_ × 7H_2_O, 0.009 g/L CaCl_2_ × 2H_2_O, and 0.052 g/L KCl, and Allen trace element solution (0.91 mM MnCl_2_ × 4H_2_O, 1.18 mM Na_2_B_4_O_7_ × 10H_2_O, 0.08 mM ZnSO_4_ × 7H_2_O, 0.03 mM CuCl_2_ × 2H_2_O, 0.01 mM Na_2_MoO_4_ × 2H_2_O, 0.02 mM VOSO_4_ × 2H_2_O, and 3.56 μM CoSO_4_ × 7H_2_O) following Kölbl *et al.* (2022) with tryptone (0.1%) as N-source over a period of 96h.”

**Line 189:** Tryptone is mentioned which I understand is used as an electron donor, but Fe_2_O_3_ is not a suitable electron acceptor for this strain.

Thank you for your comment. Tryptone was used as the N-source in the cultivation of *A. manzaensis*. It is correct that solid Fe_2_O_3_ is not a suitable electron acceptor for direct bioleaching of *A. manzaensis*. However, the ESA basalt analog was pretreated by milling into a fine powder to break the crystalline matrix and increase the surface area to accelerate the chemical dissolution rates. Furthermore, the pH was adjusted using H_2_SO_4_, which dissolved Fe_2_O_3_ into soluble Fe^3+^.

Fe_2_O_3_ (s) + 3H_2_SO_4_ (aq) → Fe_2_(SO_4_)_3_ (aq) + 3H_2_O

Therefore, owing to acid solubilization at low pH, Fe^3+^ is available for indirect bioleaching of *A. manzaensis*.

**Line 232:** I would again clarify here if this culturing step was done with just the analogue material or if it was also supplemented with media. If it is the later, I would change the wording to “successful cultivation in the presence of analog material…”

Thank you for your comment; we have modified the text accordingly.

**Figure 3:** I would add one column for a growth control if you have the data (or even if there is data online). A cell count for a sample not exposed or dessicated, just subcultured, as a comparison or even the Fc set which feels like is the baseline control for every other condition.

Thank you for your comment. The Fc set was added to **Figure 3** as a baseline control.

**Figure 3. Bioreactors, cell-mineral deposition in metal exposure wells, and re-cultivation of desiccated cells. a)** Bioreactor setup of *A. manzaensis* grown on ESAE-01 basalt analog material. **b)** Box plot of successful re-cultivation of *A. manzaensis* cells in triplicate sets (A_1–3_ and B_1–3_) after one month of desiccation compared to fresh culture (Fc). The median is shown as a black line, and the mean of the samples is indicated by a plus sign (+). Eight data points per sample were obtained, corresponding to cell counts from t_0h_ to t_72h_ (cells/mL).

**Line 260:** Consider copying or moving these values to the methods section (line 167).

Thank you for your comment; we have modified the text accordingly.

**Line 281:** Is there no significant difference between the two triplicate sets of desiccated samples, or the desiccated samples when compared to a recultivated, non-exposed sample (e.g. Fc)? Also, if you did some kind of significance test, I would add the significance value here and consider adding a line or two about data analysis/statistics, with the type of test used, in the methods, not just the specific package or online tool.

Thank you for the suggestion; we have applied a single-factor ANOVA to both re-cultivation experiments of desiccation and exposure. We have added the following description in the Materials and Methods section, Line 244: “Single-factor ANOVA analysis for determining the statistical significance of the endpoints of re-cultivation was performed using the LibreOffice extension Analysis of Variance (ANOVA) for all re-cultivated samples for the parameter cell/mL at t = 72h for triplicate sets (desiccation experiment: A_1-3_ and B_1-3_) or quadruplet sets (Fc and exposure experiment: e, ne, Lc, and Tc) with α = 0.05.”; and added for the desiccation experiment in Line 290: “The mean number of cells was between 2.5–3 × 10^6^ cells/mL throughout the two triplicate sets (A_1–3_ and B_1–3_) compared to a mean number of cells of 4–4.5 × 10^6^ in a fresh culture (Fc). When comparing the triplicate cell counts of the final cell numbers (cells/mL) upon reaching stationary phase at t = 72h, single-factor ANOVA analysis revealed no statistical difference between the two desiccated sets A_1–3_ and B_1–3_ (p = 0.96 with α = 0.05) but statistical differences between Fc, and the two sets taken together A_1–3_ and B_1–3_ (p = 0.01 with α = 0.05), or individually: Fc and A_1–3_ (p = 0.02 with α = 0.05), and Fc and B_1–3_ (p = 6.97 × 10-5 with α = 0.05). While the high p-value in Fc to B_1–3_ is due to stronger variations within the final cell numbers in B_1–3_, the non-significant difference between A_1–3_ and B_1–3_ highlights the comparability of the re-cultivations, while the significant difference between both Fc and A_1–3_ and Fc and B_1–3_ reflects the effect of desiccation on the cell-mineral mixtures.”; and for the exposure experiment in Line 329: “When comparing the quadruplicate cell counts of the final cell numbers (cells/mL) upon reaching stationary phase at t = 72h, single-factor ANOVA analysis revealed statistical difference between Fc, e, ne, Lc, and Tc (p = 0.01 with α = 0.05) and between Fc, e, and ne (p = 3.91 × 10-5 with α = 0.05) but no statistical difference between the remaining samples. This highlights the difference between the fresh culture (Fc) and the prepared samples for the exposure experiment e, ne, Lc, and Tc, as well as the strong variation between Fc, e, and ne.”

**Figure 7:** This figure is ok, but I wonder if the authors could change it for, or add a version (maybe move one to the supplemental material) where the groups are switched: samples in the X axis and quinone proportions in the legend to help visualize differences between samples instead of differences between proportions of quinones. The main aim is not to profile quinones in A. manzaensis (that is secondary) but measure the effects of the UV (that is your independent variable in this case).

Thank you for the suggestion; we have added a version of **Figure 7** with reversed axis in the supplementary material (**Supplementary Figure 1_Tentative annotation of thiophene-bearing quinones in reversed axis representation**).

**Supplementary Figure 1. Tentative annotation of thiophene-bearing quinones in reversed axis representation.** **a)** Summed peak areas of putatively annotated thiophen-bearing quinones (CQ_n:x_, SQ_n:x_, and BDTQ_n:x_) in the MS spectra: exposed (e), non-exposed (ne), transport control (Tc), laboratory control (Lc), and fresh culture (Fc). **b)** Summed peak areas of the subset of tentatively annotated thiophen-bearing quinones, including only features detected in measurements for which supporting MS/MS spectra were available.

**Line 340:** do you have a value for the penetration of UV and the thickness of the samples? If the discoloration described previously affected the entire sample, I would assume the UV did penetrate the sample, unless measured.

Thank you for your comment. We have added in Line 356: “No precise measurements were taken regarding the specific penetration depth of UVs in the tested samples; however, earlier studies suggest that a few millimeters of regolith are sufficient to significantly block harmful UV radiation (shorter than 300 nm) (Carrier *et al.* 2019).”

(Please see: <https://agupubs.onlinelibrary.wiley.com/doi/full/10.1029/2018JE005758>)

**Line 343:** it is not clear why other sample preparation procedures, like the “thin films” mentioned wouldn’t be effective. Is the volume a problem, e.g not enough cells for organic extractions?

Thank you for the question. To the best of our knowledge, following the re-cultivation experiment after desiccation by Kölbl *et al.* (2020), a “thin film” would not allow the minimum cell density to initiate culture growth during re-cultivation. Additionally, as described in Line 426: “A different sample preparation procedure might also solve this problem; however, complementary analyses, such as survival assays, would not have been possible if thin films were used in Petri dishes.”

**Discussion:** The discussion starts with a description of potential targets for biosignature detection and the differences between these molecules but the discussion of the results in figure 7 make little to no mention of the differences found, therefore the section 295-301, might need slight rewording. Not familiar with these organics, but I feel like much more can be said from the mass spec results, including statistical analysis between the proportion of different types of quinones for each sample type, and not just the presence/absence of quinones (I’ve made a point below about absolute values too). For example, SQ10 columns show a drop in the number of double bonds with exposure: higher proportion of double bond SQ10 (SQ10:10 and SQ10:7) are found in samples that were not exposed compared to exposed samples (which only had SQ10 with lower number of double bonds). This could be the result of the UV breaking these bonds. Even if the control sample (Fc) lacks these, I think it is still valuable information.

Thank you for the comments and suggestions on the discussion; we have reworded and added information in the discussion starting in Line 409: “The SQ_10_ columns showed a drop in the number of double bonds with exposure: a higher proportion of double bond SQ_10_ (SQ_10:10_ and SQ_10:7_) was found in samples that were not exposed compared to exposed samples (which only had SQ_10_ with a lower number of double bonds). This could be the result of UV radiation breaking these bonds. However, while the laboratory control (Lc) did not exhibit a drop in the proportion of double bonds, the control sample (Fc) did not exhibit these characteristics. Certain indicative features, CQ_9:5_, SQ_7:4_, and SQ_8:0_, were only found in the samples extracted immediately after cultivation. SQ_10:2_ and SQ_10:5_ were present in the exposed samples and the transport control, whereas SQ_10:7_, SQ_10:10_, BDTQ_4:1_, and BDTQ_10:0_ were absent in the exposed samples. This poses a challenge when ascribing features to actual exposure. The analyzed samples were organic extracts of homogenized material immediately analyzed after exposure to resolve minor changes in the thiophene-bearing quinone headgroups visible by energetic fragmentation using MS/MS. However, the generally low yield of the corresponding MS/MS spectra (**Figure 7B**) complicates this approach, requiring further optimization.”

Are the proportions of total extracted organic compounds, cell numbers or sample weight used to normalize the proportion of quinones between samples? I couldn’t find how this was done, and it could affect the proportions in figure 7, e.g. sample Fc having a higher organic yield could affect the result if it is not considered. The article referenced in the methods only deals with one sample. Maybe I’ve missed something. I understand that mass spec data is not always useful for quantifying absolute concentrations of certain organics but then it should be mentioned in the text, either the methods or the discussion. When the data is presented this way it is inferred that the proportions of organics is the result form the data. If the data is not significant enough or the authors wanted to focus just in absence/presence then it should be mentioned in the methodology and a different graph considered, such as a heatmap or a table.

Thank you for your comments. We have clarified in the methodologies the semi-quantitative character of the MS analysis, which was calibrated throughout all measurements by lock mass calibration using an ESI-TOF tuning mix.

If you are considering carrying on with these experiments in the future, survival assays could be done with replicating stamps (to recover only the top layer), or protein/ATP assays, qPCR, transcriptomics, live/dead stains, etc. Part of the sample was divided using a sterile scalpel so I’m guessing the number of runs or access to the martian reactor was limited but it might be useful to either subdivide the sample holders themselves or use the same scalpel to separate top/bottom layers, even if just to see differences in cell counts. I think the discussion about why the UV exposure wasn’t effective should be expanded with one or two sentences and maybe a reference. I think the article needs to clarify whether figuring out if shielding of the organics by the martian basalt is the main aim of the experiments or if it is the stress on cells yields different proportions of organics. The introduction makes it seem it is the former but the experimental plan is much more similar to the later. The authors discussed a future experiment with just the organics which make me lean towards thinking the aim was to see the response on the cells, in which case, growing them with martian regolith is appropriate but then the cells could have been washed (e.g. through centrifugation and fresh media or PBS) or the basalt decanted to focus on the effect of UV on the cells. I think there needs to be on of the two experiments before this one to get a better understanding of what is happening.

Thank you for your comments and suggestions for future experiments and on the discussion; we have reworded and added information in the discussion to better clarify the role of UV exposure, as stated in a previous comment (Line 356): “No precise measurements were taken regarding the specific penetration depth of UVs in the tested samples; however, earlier studies suggest that a few millimeters of regolith are sufficient to significantly block harmful UV radiation (shorter than 300 nm) (Carrier et al. 2019).”, and the study objectives starting in Line 366: “Similar potential biosignificant target sites for molecular alterations have been reported for lipid biomarkers by Finkel *et al.* (2025), including unsaturation, methyl groups, and/or polycyclic ring structures, identifying saturated chains with functional groups as the “most reliable biomarkers for long-term preservation”, especially when embedded and thus shielded from radiation in appropriate mineral matrices (Finkel *et al.* 2023). Therefore, using cell-mineral mixtures and after an initial desiccation experiment to establish the necessary methodology for molecular analysis and re-cultivation techniques, an exposure experiment under Mars-like conditions was conducted to test not only the resilience of the microbial cells to Mars-like conditions but also to further elucidate the role of these molecules as potential biosignatures for life on Mars and other celestial bodies.”

I would like to see a mention about oxygen in the discussion or the methods. The organism selected is grown anaerobically following the protocol from Yoshida *et al.* 2006 but air desiccated. The introduction also mentions how the presence of oxygen during growth of other species affects the proportion of quinones. I would include what considerations were undertaken to deal with oxygen contamination or how it was handled a posteriori statistically. In the future there might need to be an oxygen/no oxygen control. Similarly controls could have been carried out for isolating and measuring the effects of temperature.

Thank you for your comments. We have clarified in the manuscript that the microorganisms were grown aerobically under a stream of CO_2_ and were therefore continuously exposed to oxygen throughout cultivation and air desiccation. However, we are thankful for the suggestions and will implement them in future purely anoxic experiments.

**Reviews of manuscript FEMSMC-2025-082.R1**

**Reviewer 1: Mara Leite**

**Recommendation: Accept**

I thank the authors for their thorough response and for carefully addressing all of my previous comments. The manuscript is stronger and clarifies and addressed the language and clarity issues raised in the previous round.

**Reviewer 2: Alvaro Del Moral, The Open University**

**Recommendation: Accept**

The authors have made the appropriate changes and the main issues I had (regarding the methods and discussion) have addressed. I would still strongly suggest swapping figure 7 for the one in the supplementary material. The new one looks good and I think it is far easier to read and deduce the main effects of the desiccation and radiation on the proportion of quinones. However, as long as the new figure is present somewhere then I am happy for the paper to be published. I leave it to the authors to decide what to do with it.

**Associate Editor Recommendation: Accept**

**EIC Decision: Accept**
