# Supplementary material for: Assessing cell survival and biosignature preservation of the extremely thermoacidophilic archaeon Acidianus manzaensis after exposure to Mars-like conditions
Source: FEMS Microbes. 2026 Jul 30;7:xtag043. doi: 10.1093/femsmc/xtag043 (PMC13471030; doi:10.1093/femsmc/xtag043)
Supplement: Supplementary file 1 [file xtag043_supplemental_files.zip › Gfellner et al (2026) Supplementary Figure S1_Tentative annotation of thiophene-bearing quinones in reversed axis representation.docx]

**Supplementary Figure 1. Tentative annotation of thiophene-bearing quinones in reversed axis representation. a)** Summed peak areas of putatively annotated thiophen-bearing quinones (CQ_n:x_, SQ_n:x_, and BDTQ_n:x_) in the MS spectra: exposed (e), non-exposed (ne), transport control (Tc), laboratory control (Lc), and fresh culture (Fc). **b)** Summed peak areas of the subset of tentatively annotated thiophen-bearing quinones, including only features detected in measurements for which supporting MS/MS spectra were available.
